# Use of 6 Nucleotide Length Words to Study the Complexity of Gene Sequences from Different Organisms

**DOI:** 10.3390/e24050632

**Published:** 2022-04-30

**Authors:** Eugene Korotkov, Konstantin Zaytsev, Alexey Fedorov

**Affiliations:** 1 Institute of Bioengineering, Federal Research Center of Biotechnology of the Russian Academy of Sciences, 119071 Moscow, Russia; 2 Bach Institute of Biochemistry, Research Center of Biotechnology of the Russian Academy of Sciences, 119071 Moscow, Russia; con.zaytsev@gmail.com (K.Z.); a.fedorov@fbras.ru (A.F.)

**Keywords:** cds, genome, bacteria, plants, metazoa, Gini coefficient

## Abstract

In this paper, we attempted to find a relation between bacteria living conditions and their genome algorithmic complexity. We developed a probabilistic mathematical method for the evaluation of k-words (6 bases length) occurrence irregularity in bacterial gene coding sequences. For this, the coding sequences from different bacterial genomes were analyzed and as an index of k-words occurrence irregularity, we used W, which has a distribution similar to normal. The research results for bacterial genomes show that they can be divided into two uneven groups. First, the smaller one has *W* in the interval from 170 to 475, while for the second it is from 475 to 875. Plants, metazoan and virus genomes also have *W* in the same interval as the first bacterial group. We suggested that second bacterial group coding sequences are much less susceptible to evolutionary changes than the first group ones. It is also discussed to use the *W* index as a biological stress value.

## 1. Introduction

For some time, genetic information has been generated exponentially along with the development of sequencing technologies [1]. Thereby, the role of mathematical methods and algorithms, which can be applied to nucleotide and amino acid sequences research, increases. In this sense, the development of such methods is really important, as it allows to obtain new information about genomes and individual gene structures. In the last years, there was progress in mathematical methods for studying base correlations in nucleotide sequences. These methods can be divided into two groups. The first one includes spectral methods, such as Fourier transformation [2,3,4], wavelet analysis [5] and information decomposition [6]. All of them can be used to search for different length correlations in the DNA sequences having more than 2.0 substitutions per nucleotide [7]. However, the limiting factor for them is the fact, that they are very sensitive to nucleotide insertions and deletions. Insertions and deletions are common mutations in the DNA sequences of different origins [8]. There are also mathematical methods developed specifically for accounting for insertions and deletions using dynamic programming [9]. Some examples are: TRF [9], Mreps [10], TRStalker [11], ATRHunter [12], T-REKS [13], IMEX [14], CRISPRfinder [15], SWAN [16] and tandem repeat search tools, reviewed in [17].

Previous research studies allowed us to find different length periodicity in eukaryotic and prokaryotic genomes [3,8,18,19]. Three base periodicity is the most common in both eukaryotic and prokaryotic genomes [20]. The second most common is the two base periodicity, which usually occurs in noncoding regions [21]. Three base periodicity occurs in protein coding regions. Its origin is due to several factors, first of all, amino acids are not equiprobable in proteins, also genetic code is degenerative and finally, synonymous codons are not equiprobable in their use in genes. At the same time, triplet periodicity is different for genes from different genomes [22]. That is why triplet periodicity can be seen as a feature, which corresponds to the organism's adaptation to a certain environment, as well as gene and protein resistance to base substitution. If all the mutational substitutions were possible in genes, triplet periodicity would be absent. So, triplet periodicity can be used as a feature for genome classification [22]. For every studied genome an analog to the Gini coefficient (W), which is commonly used for economic inequality measurement, can be calculated [23].

However, here we could estimate inequality using triplets or any *k*-words, which are multiples of three bases. Then, having W scores for each genome, we could classify the studied genomes by the W index. It is important to note, that by using this method, *k*-word frequencies are ranked in ascending order, so their original order has no effect on *W*.

In this study, we used *k* = 6. Such a length choice was made because of several factors. Firstly, k should be proportional to three bases, so that triplet periodicity could be included in the words without any phase shifts. It is also desirable to select the largest *k* possible so that most of the correlations could be taken into account. Finally, the k value is limited by the genome size, so for the sake of statistical significance, it could not be too large. Because the size of bacterial genomes does not exceed several millions of bases and the number of words for k=9 is 262144, some of them could not be seen in the coding sequences due to the small sample effect. That is why we selected k=6 for this study.

In this study, we developed a probabilistic mathematical method for the evaluation of k-words (six bases long) occurrence irregularity in bacterial genomes coding sequences. We used the Monte Carlo method for the probabilistic estimates. All of the coding sequences from different bacterial and other organisms’ genomes were analyzed and the *W* index of k-word occurrence irregularity for them was calculated. *W* has a distribution similar to normal. *W* statistical significance was estimated using the Monte Carlo method. Such calculations were made to find a relation between bacteria living conditions and their genome complexity. Genome complexity is seen as an algorithmic complexity (Kolmogorov complexity [24]) of a gene. For a random DNA sequence, it would be similar to the sequence length and vice versa. If for example the sequence is composed only of {att}*_n_* subsequences, then the algorithmic complexity for this sequence would be slightly larger than 0. In this sense, the larger the *W* value, the lesser the genome algorithmic complexity is. There is no effective method for calculating algorithmic complexity [25], so we used *W* for its estimation.

The research results for bacterial genomes show that they are divided into two uneven groups. The first, the smaller one, has *W* in the interval from 170 to 475, while for the second it is from 475 to 875. This shows that the second group maintains a much higher irregularity level of k-word occurrence than the first group. Additionally, algorithmic complexity [24] for the first group is much higher than for the second one.

It can also be seen, that six-word occurrence irregularity in bacterial, metazoan and plant cds is mainly due to the triplet periodicity. However, triplet correlation contribution to the six-word occurrence irregularity is the highest in bacterial genomes. Generally, the six-word occurrence irregularity is much higher in bacterial genomes, than in the genomes of plants and metazoa. Based on the results, it could be suggested, that the coding sequences from the second bacterial group are far less sensitive to evolutionary changes than the ones from all the other organisms studied.

## 2. Materials and Methods

### 2.1. DNA Sequences

Prokaryotic gene coding sequences, used in this study were taken from http://bacteria.ensembl.org/index.html (accessed on 27 September 2021). For the calculations, only one strain for each bacterial species was used. In total, we used 9236 bacterial genomes. Plant gene coding sequences were taken from http://plants.ensembl.org/index.html (accessed on 5 October 2021) and metazoan gene coding sequences were taken from http://metazoa.ensembl.org/index.html (accessed on 5 October 2021). Virus gene coding sequences were taken from ftp server ftp://ftp.ncbi.nlm.nih.gov/genomes/Viruses (accessed on 11 October 2021). In total 70 plant species, 73 metazoan species and 54718 virus species were used. When selecting genomes, for each species only one strain was taken to avoid overrepresentation.

### 2.2. W Calculation Algorithm

For *W* calculation we filled a 4096 size U array for each coding sequence of the studied genome. If S is cds, then s(i) is the numerically encoded cds, where 1 = a, 2 = t, 3 = c, 4 = g. Such encoding was made just to ease computation. We started with *i* = 1 *s*(*i*) sequence for every cds and calculated:

(1) $$j=s(i)+\sum_{k=i+1}^{i+5}\left(s(k)-1\right)4^{k-i}$$

Where i is in the {1,4,7,…,l−5} series. Here, l is the length of S sequence. For every calculated j we added one to the corresponding *U* array cell: *U*(*j*) = *U*(*j*) + 1. This means that we calculated a number of six-words with three base steps. So, every six-word intersected the previous one by three bases. Three base shifts were selected as the least possible not to interrupt triplet periodicity [20], but we still could consider six-words generated in both phases. Here, is an example. Let *S* = {atgtagctgactgta} and step length is six bases. Then in the first phase, there are atgtag, ctgact words, and in the second phase with a three bases shift there are tagctg actgta words. If we calculate the number of six-words with three bases, there are atgtag, tagctg, ctgact and actgta words, which is a sum of words in both phases.

These calculations were made for all the coding sequences of a single studied genome. After *U* array filling, it was normalized by 10^6^. The sum of the array:

(2) $$Sum=\sum_{k=1}^{4096}u(k)$$

was calculated. Here, *u*(*k*) is an element of the U array. Next, the Q array was calculated for each j=1,…,4096 as $q(k)=10^{6}u(k)/Sum$. q(k) is an element of the Q array. Such normalization is needed to eliminate the set size influence on the array. Then, the Q array was sorted in ascending order. The resulting array was named $Q_{1}$. Such procedures are similar to those used in Gini coefficient calculation [23] and when applying Zipf’s law to *k*-words bp from different DNA sequences [26,27,28].

Next, we calculated R and T arrays using the Monte Carlo method. In the case of *R*, coding sequences were mixed randomly and it was made in a way that no stop codons would be generated in the mixed sequences. Then we used the (1) formula and filled R array the same way as Q. For each studied genome each cds was mixed 100 times to reduce the statistical fluctuation influence on R. After that every element *R*(*j*), *j* = 1, 2, ..., 4096 was divided by 100.

T array is calculated the same way as R, but this time cds were mixed in triplets instead of single bases. This means that codons in cds remained the same, but their order was changed. All the other procedures were the same as for the *R* array.

After *R* and *T* were calculated, we sorted them in ascending order, the same as for Q array. The resulting arrays were named *R*_1_ and *T*_1_ accordingly. Then we determined the difference between *Q*_1_ and *R*_1_ distributions. For that matrix *M*(2,4096) was filled: $M(1,i)=Q_{1}(i)$ and $M(2,i)=R_{1}(i)$ for *i* = 1, ..., 4096. Next, we calculated *I*:

(3) $$I=\sum_{i=1,}^{2}\sum_{j=1}^{4096}m(i,j)\ln m(i,j)-\sum_{i=1}^{2}x(i)\ln x(i)-\sum_{j=1}^{4096}y(j)\ln y(j)+L\ln L$$

Where $x(i)=\sum_{j=1}^{4096}m(i,j)$, $y(j)=\sum_{i=1}^{2}m(i,j)$ and m(i,j) is an element of *M* matrix. *L* is the sum of all *M* matrix elements. This is the mutual information formula for considering correlations between rows and columns features [29]. *p*(*i*,*j*) = *m*(*i*,*j*)/*L*, *p*(*i*,*) = *x*(*i*)/*L*, *p*(*,*j*) = *y*(*j*)/*L*. As in [29] we are checking two hypotheses. Hypothesis *H*_1_ is that *p*(*i*,*j*) ≠ *p*(*i*,*) *p*(*,*j*) and hypothesis *H*_2_ is that *p*(*i*,*j*) = *p*(*i*,*) *p*(*,*j*). Then, *I* is mutual information for discrimination for *H*_1_ against *H*_2_. This means the more difference between *m*_1_(1,*j*) and *m*_2_(1,*j*), *j* = 1,2,...,4096, the greater *I* is. 2*I* can be considered as a random value having $\chi^{2}$ distribution with 4095 × 15 degrees of freedom [29]. We calculated the normal distribution argument $W_{1}=\sqrt{4I}-\sqrt{2*4095*15-1}$. Such approximation of $\chi^{2}$ distribution to normal distribution works well in the *W*_1_ range from −10.0 to 1500.0. That is enough for bacterial genomes and other species research. The full *W*_1_ value range depends on *L*. In our case *y*(1) = *y*(2) = 10^6^, and therefore, *L* = 2 × 10^6^. For such *L* values, minimum *W*_1_ = −89.7, while the maximum possible value is about 7400. This value can be obtained only theoretically if, for example, in *Q*_1_ there would be only four nonzero cells (for instance, there would be only a (*a*)_6_, (*t*)_6_, (*c*)_6_, (*g*)_6_ six-words). The greater the value *W*_1_ is, the lower the probability of *Q*_1_ and *R*_1_ being different due to random factors. If *Q*_1_ and *R*_1_ are identical, then *I* is zero (*W*_1_ = −89.7). 

We have also determined the normal distribution argument *W*_2_, which allows estimating the difference between *Q*_1_ and *T*_1_ arrays. All the calculations were the same as for *W*_1_, but the *T*_1_ array was used instead of *R*_1_. *W*_2_ has approximately the same distribution as *W*_1_. We calculated *W*_1_ and *W*_2_ for all genomes listed in Section 2.1. 

**Figure 1:**
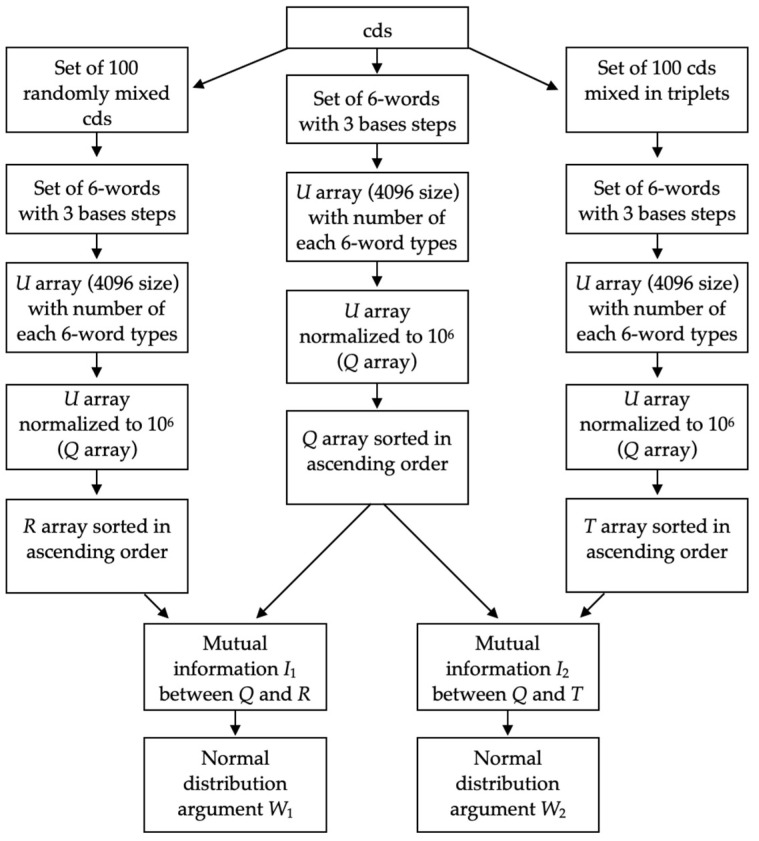
Structure of the algotithm for normal distribution arguments W1 and W2 calculation from cds for each species.

Schematic representation of the algorithm is shown in Figure 1.

It is important to note, that triplet order in *M*(1,*j*) and *M*(2,*j*) (*j* = 1,2,...,4096) arrays can be totally different. At the same time, each of these arrays is sorted in ascending order. This lets us avoid local unevenness impact on *W*_1_ and *W*_2_ when studying different genomes. For instance, if we take unsorted expected frequencies as an *R*_1_ array and use them in the *M*(2,*j*) string, then local unevenness may have a strong impact on *W*_1_ and *W*_2_. Here is an example, let there be a gene, composed of 300 codons (900 nucleotides) with a, t, c and g frequencies being 0.3, 0.1, 0.3 и 0.3, respectively. Let a, c and g bases be randomly dispersed along the gene sequence. Additionally, let all 90 t only be found in a row (ttt…t). Then, the number of tttttt six-words would be 29. The total number of six-words, that can be found with 3 bases step is 299. The expected number of tttttt words can be estimated as 299 × (0.1)^6^ ≈ 3 × 10^−4^. *Z* is a normally distributed observable number of these six-words deviations from the estimated number. For its calculation, a normal approximation for binomial distribution is used. Z = (29 – 3 × 10^−4^)/{(3 × 10^−4^)(1 − 10^−6^)}^0.5^ ≈ 1700. 3 formula is an informational analog to χ^2^ distribution used for theoretical and experimental distributions comparison [29]. This way, these six-words contributions to *I* in the (3) formula will be significant. However, as the rest of the sequence is random, the use of unranged expected frequencies in *R*_1_ will lead to a significant undervaluation of such gene algorithmic complexity. That is why six-words sorted in the ascending order were used in *M*(2,*j*).

## 3. Results

### 3.1. Comparison of Q_1_ and R_1_ Arrays with Q_1_ and T_1_ Arrays

**Figure 2:**
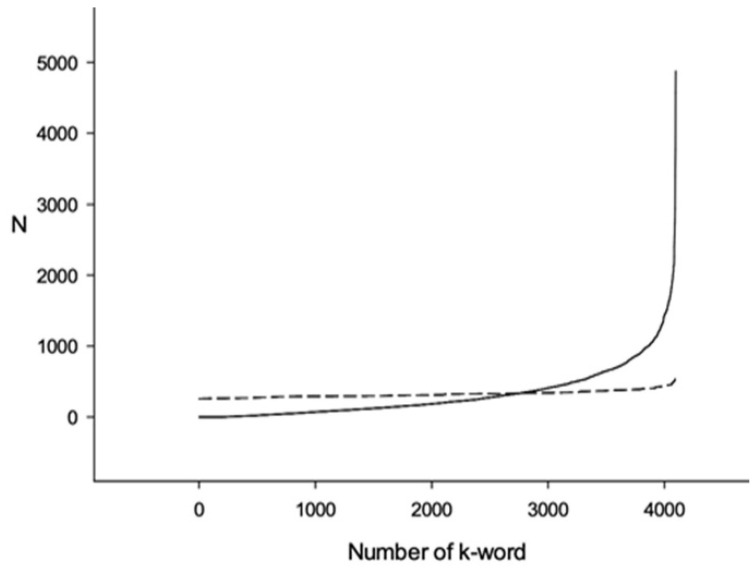
Six-word distribution sorted in ascending order. Continuous line shows $Q_{1}$ distribution for *E. coli* genome cds. Dotted line shows $R_{1}$ distribution for the same genome after every cds was randomly mixed. Mixing was performed in a way, that no stop codons can be found in resulting sequences.

**Figure 3:**
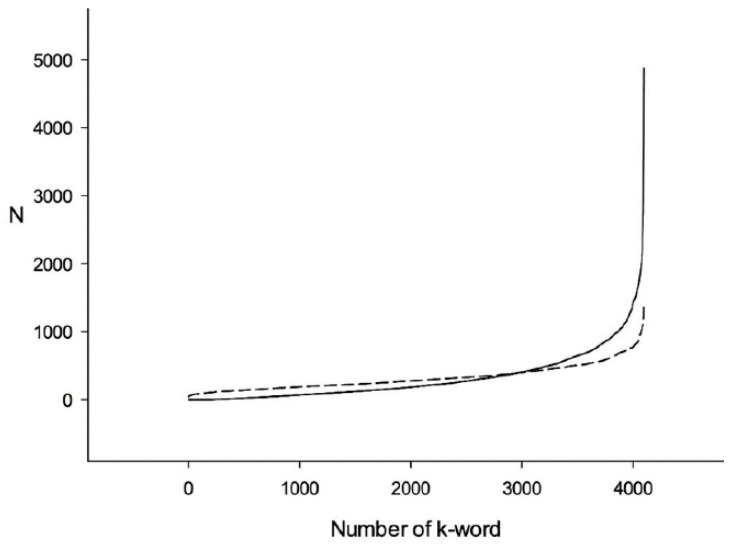
Six-word distribution sorted in ascending order. Continuous line shows $Q_{1}$ distribution for *E. coli* genome cds. Dotted line shows $T_{1}$ distribution for the same genome after every cds was mixed by triplets.

An example of resulting arrays *Q*_1_ and *R*_1_ for the *E. coli* genome is shown in Figure 2 (continuous line for *Q*_1_ and dotted line for $R_{1}$). It can be seen that there is a distinct difference between them. An example of *Q*_1_ and *T*_1_ arrays is shown in Figure 3. It is obvious that there is much less difference between *Q*_1_ and *T*_1_ than between *Q*_1_ and *R*_1_ arrays. That is because the difference between *Q*_1_ and *R*_1_ is due to triplet cds periodicity as well as the correlation between triplets in every six-word, while all the difference between *Q*_1_ and *T*_1_ is only due to the correlation between neighboring triplets.

Here is an example, if *S*_1_ = {*atc*}_100_ is a sequence, containing only an *atc* triplet repeated 100 times, then the *atcatc* six-word appearance is only due to triplet periodicity. This can be demonstrated with a little math. The *Sum* for this sequence, calculated using (2) formula, is 99 and *p*(a) = *p*(t) = *p*(c) = 1/3, *p*(g) = 0. *S*_2_ is the sequence we can obtain if we were to shuffle the *S*_1_ sequence randomly. Then the probability of *atcatc* word appearance can be calculated as *p*(*atcatc*) ≈ *p*(*a*)^2^*p*(*t*)^2^*p*(*c*)^2^ = (1/3)^6^ ≈ 0,0014. There are Sum*p(*atcatc*) such words on average in the *S*_2_ sequence. The normal distribution argument can be calculated as:

(4) $$Z=\frac{\left(N-Sum*p\right)}{\left(Sum*p{\left(1-p\right)}^{0.5}\right)}$$

Here, *N* = 99 is the number of *atcatc* words in the *S*_1_ sequence when searched with a step length of three bases, *p* = *p*(*atcatc*). *Z*_12_ ≈ 267 is the normal distribution argument, showing a six-word frequency deviation between *S*_1_ and *S*_2_ sequences. This means that the probability of obtaining 99 *atcatc* words in a row is *P*(*x* > 267), where *x* is a normally distributed random variable. It is an extremely low value. At the same time, if we were to create an *S*_3_ sequence by mixing *S*_1_ in triplets, for such sequence *p* = *p*(*atcatc*)=1 and calculated by the formula (4) normal distribution argument Z_13_=0. So, this example shows that when shuffling the *S*_1_ sequence in triplets, there is no effect of triplet periodicity on the six-words frequency. This means, that for the *S*_1_ = {*atc*}_100_ sequence, *Q*_1_ and *R*_1_ distributions would be different and *Q*_1_ and *T*_1_ would be identical.

For the next example, *S*_4_ = {*tttccc*}_50_, consisting of *tttccc* word repeated 50 times. Here, *p*(a) = *p*(t) = 0.5, *p*(c) = *p*(g) = 0. Sum=99 for such sequence ((2) formula), *N*=50 for *tttccc* word. *S*_5_ is the randomly mixed sequence S _4_, and for *S*_5_ *p* = *p*(*tttccc*) ≈ (0.5)^6^ ≈ 0.016. So, there are Sum*p ≈ 1.6 such words on average in the *S*_5_ sequence. The normal distribution argument calculated by the (4) formula is *Z*_45_ ≈ 38. The *S*_6_ sequence is created by shuffling the *S*_4_ sequence in triplets. Here*, p* = *p*(tttccc) increases because there are only four possible types of six-words: *tttccc*, *cccttt*, *cccccc* и *tttttt*. So, *p* = *p*(tttccc) and the normal distribution argument *Z*_46_ = 5.6 as calculated by (4) formula. As a result, $Q_{1}$ and $R_{1}$ distributions are different (*Z*_45_ ≈ 38) for *S*_4_ = {tttccc}_50_ sequences, but *Q*_1_ and *T*_1_ are also different (*Z*_46_ ≈ 5.6). The reason for this is that in addition to quite an evident triplet periodicity, sequence *S*_4_ has a six-word periodicity. At the same time, the *S*_1_ sequence only has a three base periodicity, which is fully taken into account when mixing the sequence *S*_1_ in triplets, since *Z*_12_ ≈ 267 and Z_13_ ≈ 0.

This example shows that when mixing is performed randomly, there are three nucleotide long words contributing to *W*_1_ as well as longer than three bases words (6, 9, 12, ... nucleotides long). When shuffling in triplets, only six nucleotides and longer correlations can affect *W*_2_. Such phenomena can be seen when comparing Figure 2 and Figure 3, which were computed for the *E. coli* genome. Here, the differences between *Q*_1_ and *T*_1_ are much smaller than between *Q*_1_ and *R*_1_. Presumably, the *Q*_1_ distribution irregularity is partially due to triplet periodicity and partially due to triplets’ correlation in six-words. *W*_1_ and *W*_2_ for the *E. coli* genome are 799.13 and 439.11, respectively. These values show that the six-words distribution irregularity in the *E. coli* genome is due to both the triplet periodicity presence and the triplets’ correlation in six-words.

### 3.2. $W_{1}$ and $W_{2}$ Distributions for Bacterial, Metazoan and Virus Cds

**Figure 4:**
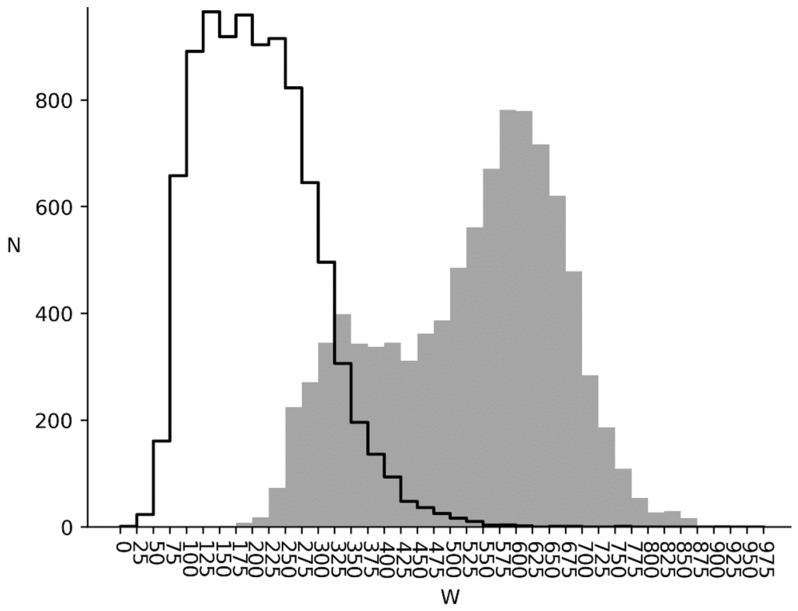
Bacterial genomes. Grey infilled area is *W*_1_ distribution for bacterial genomes. Black outlined area is *W*_2_ distribution for bacterial genomes.

$W_{1}$ and $W_{2}$ distributions for bacterial genomes are shown in Figure 4. It can be seen, that *W*_1_ and *W*_2_ are greater than zero, which shows, that correlation between nucleotides is present for all bacteria studied. The *W*_1_ distribution is shown as a grey area and the $W_{2}$ is shown as an area with a black outline. It can be seen, that the *W*_1_ distribution has two peaks. The first one is located in *W* = 325 range and the second one is in *W* = 600 range. As can be seen, bacterial genomes can be divided into two groups. The first of them has *W*_1_ between 175 and 425 and the second is between 425 and 875. There is a slight irregularity in the six-word distribution for the first group relatively to mixed sequence (*W*_1_). For the second group, the difference is much larger.

It should be also mentioned, that when switching from *W*_1_ to *W*_2_ there is a significant change in distribution form. Instead of two peaks, as seen before, there is only one in the *W*_2_ = 200 range. Such behavior is due to six-word distribution irregularity being mostly subject to triplet irregularity in the cds instead of triplet correlation.

**Figure 5:**
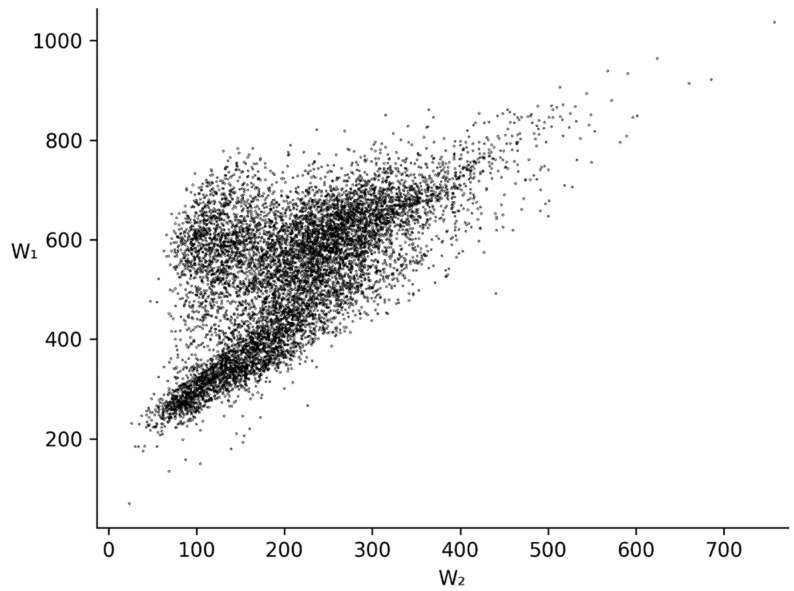
The scatter plot for *W*_1_ and *W*_2_.

We have also made a scatter plot for *W*_1_ and *W_2_* for all bacteria genomes studied, which is shown in Figure 5. Here, three clusters can be seen. The first *W*_1_ peak between 175 and 425 from Figure 4 is transformed into an elongated cluster with a center at (*W*_2_, *W*_1_) ≈ (135, 320) in Figure 5. The second *W*_1_ peak from Figure 4 is transformed into two clusters in Figure 5. The first one has its center at (*W*_2_, *W*_1_) ≈ (120, 600), and the second one is at (*W*_2_, *W*_1_) ≈ (220, 610). The first cluster has a high triplet periodicity impact on six-word frequency, while for the second one this impact is much lower, but it is still there.

**Figure 6:**
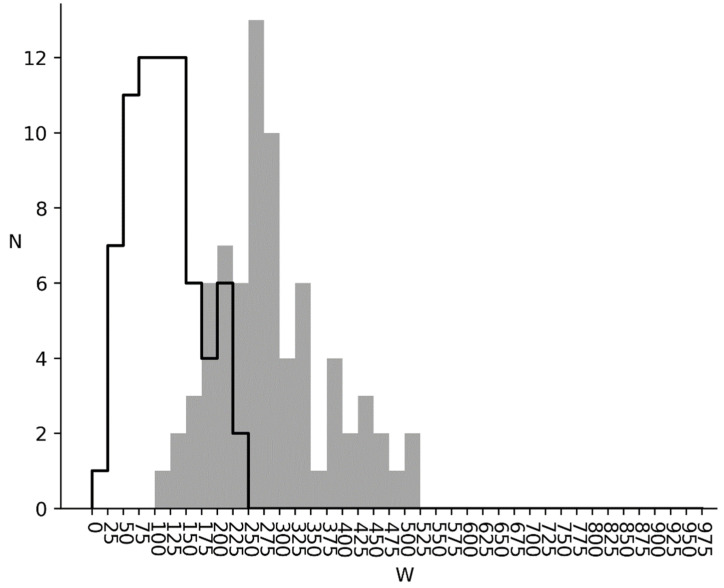
Metazoan genomes. Grey infilled area is $W_{1}$ distribution for metazoan genomes. Black outlined area is $W_{2}$ distribution for metazoan genomes.

*W*_1_ and *W*_2_ distributions for metazoan genomes are shown in Figure 6. There is only one peak for *W*_1_, located in the same region as the first bacterial group. This means, that there is much less irregularity in six-word use in metazoan cds, than in the bacterial ones. 

The second peak from Figure 4 is almost absent in Figure 6. *W*_2_ distribution in Figure 6 is also located to the left as opposed to the one for bacterial genomes. So, the irregularity in six-word use being due to triplet correlation is much lower in metazoan genomes, than in bacterial ones.

**Figure 7:**
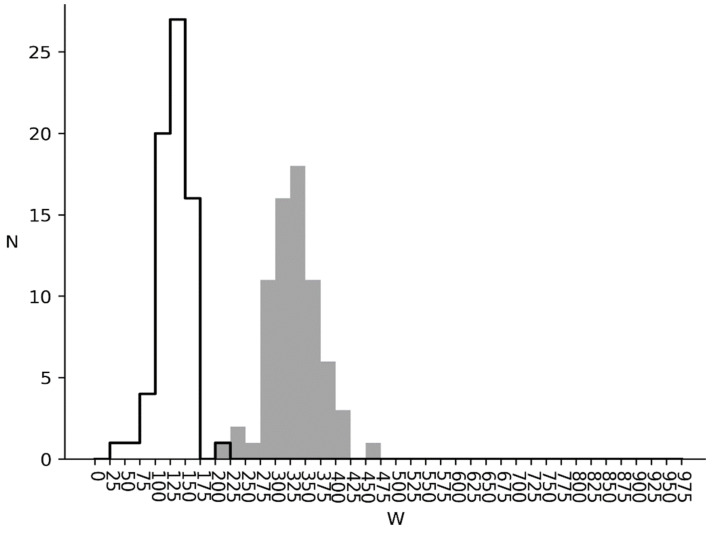
Plant genomes. Grey infilled area is $W_{1}$ distribution for plant genomes. Black outlined area is $W_{2}$ distribution for plant genomes.

The same can be seen for plant genomes’ $W_{1}$ and $W_{2}$, as shown in Figure 7. There is also only one $W_{1}$ peak and $W_{1}$ distribution for plants is located in about the same area, as the one for metazoan genomes. $W_{2}$ distribution is even more offset to the left relative to the metazoan genome $W_{2}$. All of this means, that there is quite a large triplet periodicity contribution to six-word distribution for plant genomes, while the triplet correlation is minor.

We have also analyzed all the virus genomes available. All the virus cds were combined in one set because the genomes are quite short. For this set $W_{1}=233.6$ and $W_{2}=188.7$. So, for virus cds, the six-word distribution is the closest to the one for randomly mixed sequences as opposed to other organisms studied. $W_{2}$ is not that much smaller than $W_{1}$, which means, that triplet correlation is quite significant and triplet periodicity is not the main factor in six-word distribution irregularity.

## 4. Discussion

The K-words frequencies ranging procedure is used in the Gini coefficient [23] and when studying Zipf’s law in DNA [26,27,28]. However, Zipf’s law is that there is a quantitative relationship between a word’s rank and its frequency in the text [30]. However, the use of Zipf’s law for assessing gene algorithmic complexity seemed exigent for us. That is why in this paper we used k-words ranging as in the Gini coefficient calculation but calculated a quantitative estimate of the difference between k-words ranged frequencies and frequencies, obtained for shuffled gene sequences.

In this study *W*_1_ and *W*_2_ characterize the difference between ranged in ascending order six-word distributions in cds and random distributions obtained by mixing cds bases randomly (*W*_1_) and in triplets (*W*_2_), respectivelly. Distributions are ranged in ascending order with no respect to the specific six-word position. This kind of impersonality allows considering the six-word appearance irregularity in cds as a specific genome feature, representing genetic text information redundancy. The greater *W* is, the greater cds information redundancy is with a simultaneous decrease in information volume, which can be calculated by Shannon formulas [31]. The greater text redundancy, the more mutations are needed to distort the original meaning.

Previously, informational redundancy has been studied for European languages (including Russian) and it turned out that their redundancy exceeds 50% [32,33]. Some special tests conducted for the English language by Shannon [34] showed that missing letters recovery can be made only if their number does not exceed 25% of the text length. When the text reduction rate is higher, the original meaning cannot be recovered as the text becomes a meaningless set of letters, based on which it cannot be imagined, what the original point was. Simply speaking, informational redundancy shows the percentage of excess symbols (letters, words, etc.). In a text with 0 informational redundancy no error can be fixed without a meaning loss.

Considering the results of studies [32,33] and study [34], the second group of bacteria with *W*_2_ between 425 and 875 have cds with a high level of informational redundancy. We can suggest that this redundancy is needed for better genome noise immunity. In this sense, metazoan and plant cds noise immunity is much lower (Figure 6 and Figure 7). Virus noise immunity is about at the same level as the metazoan and plants one (*W*_1_ = 233.6 and *W*_2_ = 188.7). The biological interpretation is that the virus life cycle in a cell is quite short and there are a lot of them. In this condition, noise immunity is not the key factor for virus survival, but genome volatility and new virus strain creation ability are.

We attempted to compare the bacteria from both sides of the *W*_1_ spectrum shown in Figure 4. The first 10 bacteria with the highest *W*_1_ are listed in Table 1. For most of these bacteria, their habitat is limited to mammalian intestinal microbiota or the oral cavity. For example, *Alysiella filiformis* habitat [35] is mostly limited to the animal oral cavity. The same is true for *Elusimicrobium*_sp_an273 [36], *Moraxella caviae* [37], *Alysiella crassa* [38], *Kingella kingae* [39], *Acidaminococcus* sp cag_542 [40], *Helicobacter_ailurogastricus* [41]. *Urubureella suis* [42] was isolated from the heart and lungs of pigs with pneumonia and pericarditis. *Moraxella atlantae* [43] was isolated from a female cancer patient with aerobic blood cultures. Out of all the bacteria in Table 1 only Herpetosiphon geysericola [36] is unrelated to mammals and is an extremophile. It was isolated from the biofilm of a hot spring in lower California, Mexico. This organism is able to live in extreme environments, such as extreme temperature, radiation, salinity or pH levels [44].

It is important to note, that all the bacteria in Table 1 are gram-negative. Due to stronger and less permeable cell walls, gram-negative bacteria are more resistant to antibodies and live under stress than gram-positive bacteria [45].

Next, let us have a look at Table 2. Here, 10 bacteria with the lowest *W*_1_ value are listed. *Rickettsiales bacterium* [46] has the lowest *W*_1_ value and it was isolated from the south part of the Atlantic ocean. Its life cycle consists of two stages: vegetative and resting. The resting form of *Rickettsiales* is a spherical still cell, located in arthropod and warm-blooded organisms’ cells. Their reproduction happens only in live calls, similar to viruses. In the resting stage *Rickettsiales* bacterial cells are not affected by any actions from their carrier. Then there are some bacteria from the Archaea domain in Table 2. Such bacteria are *Lokiarchaeum*_sp_gc14_75 [47], *Nitrosopumilales_archaeon* [48] and *Candidatus nitrosocosmicus franklandus* [49]. Additionally, there are groups of bacteria living in water and soil. Such examples are *Sulfurovum*_sp. [50], *Cryomorphaceae bacterium* [51], *Alkaliphilus*_sp [52], *Verrucomicrobiales_bacterium* [53], *Legionellales_bacterium* [54], *Puniceicoccaceae_bacterium* [55]. It can be suggested with enough confidence that these bacteria are living in a natural environment for a long enough evolutionary time and their level of environmental stress is at a minimum.

There are almost equal amounts of both gram-positive and gram-negative bacteria in Table 2. That is not surprising, as their living conditions are less stressful and a strong cell wall presence is not an essential condition for survival.

In Table 1, there are only extremophiles or bacteria isolated from mammals. In the latter case, bacteria have to fight against the mammalian immune system, which can be a big stress for them. On the other hand, bacteria shown in Table 2 are living in an environment with minimal stress levels. The method for *W*_1_ determination, used in this study is the modified Gini method. The only difference is that we use a probability measure for *Q*_1_ and *R*_1_ distribution differentiation, but the distributions are obtained the same way as with the Gini coefficient calculation. In economics the Gini coefficient is a social stress indicator, showing the level of wealth inequality [56]. Based on Table 1 and Table 2 we can suggest that in the case of cds it also represents stress, but this time a biological one. Algorithmic complexity [25] for bacteria from Table 1 is less than for bacteria from Table 2. This suggests that bacteria gene sequences are less complex for bacteria living in less stressful environments.

## Figures and Tables

**Table 1:** Names and accession numbers for 10 bacteria with the highest *W*_1_ value.

№	Bacterial Name	Accession Number	*W*_1_
1	*Alysiella_filiformis* dsm_16848	gca_900230205	1037.38
2	*Uruburuella_suis*	gca_004341385	964.25
3	*Elusimicrobium_sp* an273	gca_002159705	940.01
4	*Moraxella_caviae*	gca_002014985	933.66
5	*Alysiella_crassa*	gca_900445245	922.0
6	*Kingella_kingae*	gca_001458475	914.74
7	*Acidaminococcus_sp* cag_542	gca_000437815	907.44
8	*Moraxella_atlantae*	gca_001678995	893.4
9	*Herpetosiphon_geysericola*	gca_001306135	880.38
10	*Helicobacter_ailurogastricus*	gca_001282985	871.84

**Table 2:** Names and accession numbers for 10 bacteria with the lowest *W*_1_ value.

№	Bacterial Name	Accession Number	*W*_1_
1	*Rickettsiales_bacterium*	gca_002691145	134.34
2	*Sulfurovum_sp*	gca_002733355	158.68
3	*Lokiarchaeum_sp* gc14_75	gca_000986845	176.05
4	*Cryomorphaceae_bacterium*	gca_002682945	179.68
5	*Nitrosopumilales_archaeon*	gca_003856905	183.85
6	*Alkaliphilus_sp*	gca_002733545	184.78
7	*Candidatus_nitrosocosmicus_franklandus*	gca_900696045	185.51
8	*Verrucomicrobiales_bacterium*	gca_002705125	192.75
9	*Legionellales_bacterium*	gca_002719415	198.52
10	*Puniceicoccaceae_bacterium*	gca_002690565	206.6
